# Antiseptic Midwifery

**Published:** 1881-02

**Authors:** W. Paulson


### ANTISEPTIC MIDWIFERY.

THERE is, I think, no doubt that a very large proportion of deaths resulting from confinement are due to puerperal fever. To general practitioners the subject is of the greatest practical importance, since cases of midwifery, scarlet fever, erysipelas, and foul wounds, are not unfrequently under their care at the same time. A case of puerperal fever is one of burning anxiety to the attendant, both at the time and for weeks afterward, as well as of pecuniary loss. The sacrifice of a life under such circumstances is peculiarly distressing, and the subject has for years engaged my attention. The precautions I now adopt are the following, which, I am happy to say, have thus far proved effectual. After seeing erysipelas, etc., I change my clothes, and always carry with me a collapsable tube, made for me by Messrs. Richardson, of Leicester, containing a solution of thymol in firm vaseline. It should be rubbed well on to the hands when quite dry, and will then last a long time, as it is very difficult to wash off, much more so than lard or oil. These tubes are very portable and convenient, and I believe their habitual use protects both surgeon and patient from possible contagion.—*W. Paulson, L.R.C.P., in London Lancet.*

